# The association between the Dietary Inflammatory Index and breast cancer risk: an updated systematic review and meta-analysis

**DOI:** 10.3389/fnut.2026.1735685

**Published:** 2026-02-04

**Authors:** Chaoyang Wang, Qian Zhou, Liang Chen, Chao Zhai

**Affiliations:** General Medical 3201 Hospital, Hanzhong, Shaanxi, China

**Keywords:** breast cancer, Dietary Inflammatory Index, meta-analysis, nutritional intervention, risk

## Abstract

**Objective:**

This meta-analysis aimed to clarify the relationship between the Dietary Inflammatory Index (DII) and the risk of breast cancer (BC), thereby providing an evidence-based foundation for the primary prevention and secondary management of BC.

**Methods:**

A systematic search was conducted in PubMed, Embase, Web of Science, and the Cochrane Library for relevant studies published up to September 30, 2025. Observational studies (case-control and cohort) reporting effect estimates (OR/HR with 95% CI) for the association of DII/E-DII with BC risk or prognostic outcomes were included. Effect sizes were pooled using a random-effects model. Heterogeneity was assessed, and subgroup analyses along with publication bias evaluation were performed.

**Results:**

Nineteen studies (14 case-control, 5 cohort) were included. Case-control studies demonstrated a significant positive association between a higher DII and increased BC risk (pooled OR = 1.91, 95% CI: 1.50–2.42), a trend consistently observed in both premenopausal and postmenopausal women. Cohort studies further confirmed that elevated DII scores were associated with a higher risk of BC (pooled HR = 1.08, 95% CI: 1.01–1.15). Each one-unit increase in the DII was correlated with an approximate 2% rise in BC risk. Moderate heterogeneity was detected among the studies, primarily attributable to variations in DII categorization methods and population characteristics.

**Conclusion:**

The current evidence suggests that the DII is associated with BC risk, with pro-inflammatory dietary patterns linked to a higher risk of breast cancer. Promoting anti-inflammatory dietary habits, characterized by abundant consumption of fruits, vegetables, whole grains, and healthy fats, holds substantial public health importance for the prevention and management of BC.

## 1 Introduction

Breast cancer (BC) remains the most common malignancy among women worldwide, with persistently high incidence and mortality rates, posing a significant public health challenge (1). Substantial evidence indicates that environmental and lifestyle factors, particularly diet, play a crucial role in the etiology and prognosis of BC (2). In recent years, chronic inflammation has been recognized as a core mechanism in cancer development and progression (3). As a modifiable factor, diet can promote or suppress systemic inflammatory status through its nutritional components, thereby influencing cancer risk (4, 5). The Dietary Inflammatory Index (DII) serves as a quantitative measure to evaluate the capacity of an individual’s dietary intake to modulate inflammatory processes. In recent years, this instrument has been extensively utilized in epidemiological research to elucidate the connections between nutritional habits and both the development and clinical course of breast cancer (6, 7).

The development of the DII relies on empirical data regarding how specific dietary constituents influence established inflammatory biomarkers. This methodology offers a standardized approach to quantify whether an individual’s overall diet tends to promote or suppress systemic inflammation. Supporting this framework, a systematic review by Chen et al. demonstrated a positive correlation between elevated DII scores and increased breast cancer susceptibility, with this relationship being especially marked in premenopausal populations (8). Corroborating these findings, an extensive Italian case-control investigation revealed that women whose dietary patterns placed them in the highest DII quintile exhibited a substantially greater risk of developing breast cancer compared to those in the lowest quintile (9). Similarly, positive associations between DII scores and BC risk, especially for specific molecular subtypes such as hormone receptor-positive tumors, have been observed in female populations in China (10), Iran (11–13), and Korea (14). Prospective cohort studies, such as the French E3N cohort (15) and the Swedish Women’s Lifestyle and Health study (16), also support the conclusion that a pro-inflammatory diet increases BC risk in postmenopausal women. Wang et al., in the PLCO cohort study, found that maintaining an anti-inflammatory diet after diagnosis was associated with reduced BC-specific mortality (17). Furthermore, Jang et al. observed among Korean BC patients that higher post-operative DII scores were associated with increased risks of cancer recurrence and overall mortality, especially in younger, obese, and node-positive patients (18).

Although numerous studies have investigated the relationship between DII and BC risk/prognosis, the findings are not entirely consistent, and there is a lack of systematic integration of the most recent evidence. Some studies reported no significant association, or observed correlations only in specific subpopulations. Furthermore, substantial heterogeneity exists across studies regarding design, population characteristics, dietary assessment methods, and statistical models, limiting comparability and the reliability of conclusions. Consequently, the conduct of an updated systematic review and meta-analysis is necessitated. This endeavor aims to provide a comprehensive synthesis of the evidence linking the DII to breast cancer risk and prognostic outcomes, while also investigating the origins of observed heterogeneity across studies.

This study aims to systematically review and meta-analyze relevant literature published up to 2025 to assess the relationship between the Dietary Inflammatory Index and the risk of BC, thereby providing evidence-based support for the primary prevention and secondary management of BC.

## 2 Materials and methods

### 2.1 Study design

This study is a systematic review and meta-analysis conducted to evaluate the association between the DII and the risk of BC. The reporting of this study adheres to the Preferred Reporting Items for Systematic Reviews and Meta-Analyses (PRISMA 2020) statement.

### 2.2 Search strategy

A comprehensive search of multiple electronic databases, including PubMed, Embase, Web of Science, and the Cochrane Library, was conducted to identify relevant records published from database inception through September 30, 2025. The search was restricted to articles published in English or Chinese. The search methodology incorporated a combination of controlled vocabulary (e.g., MeSH terms) and keywords in the title and abstract fields. The specific search strategy employed for PubMed is provided below as an illustrative example:

(“Dietary Inflammatory Index” OR “DII” OR “energy-adjusted DII” OR “E-DII”)
AND (“breast cancer” OR “breast carcinoma” OR “breast neoplasm*” OR “mammary cancer”)
AND (“risk” OR “incidence” OR “mortality” OR “survival” OR “recurrence” OR “prognosis”)
NOT (“conference abstract” OR “review” OR “editorial”).

To ensure literature saturation, the reference lists of all included articles were manually examined to identify any additional pertinent publications that might not have been retrieved through the electronic database search.

### 2.3 Eligibility criteria

The inclusion criteria were as follows:

(1)    Study type: Observational studies (cohort, case-control, cross-sectional); studies must provide extractable effect estimates [Odds Ratio (OR), Relative Risk (RR), Hazard Ratio (HR)] with corresponding 95% Confidence Intervals (CIs).
(2)    Participants: Female individuals, regardless of race or geographical region, with a pathologically confirmed diagnosis of breast cancer.
(3)    Exposure: DII or its energy-adjusted version (E-DII). The DII/E-DII must be reported either as a continuous variable or as a categorical variable (e.g., quantiles).
(4)    Outcome measures: BC risk (HR) during follow-up. Studies reporting only on prevalence or prognostic outcomes (e.g., survival, recurrence) without incidence data were excluded from the quantitative meta-analysis.

Exclusion criteria included: animal or *in vitro* studies, commentaries, conference abstracts, studies not providing original data or where data could not be obtained after contacting the authors. For duplicate publications, the version with the most comprehensive data or the most recent publication was retained.

### 2.4 DII assessment

The DII is a literature-derived, population-based dietary tool designed to quantify the overall inflammatory potential of an individual’s diet. Its development and calculation are based on a standardized global methodology, as detailed by Hébert et al. (6). The calculation of DII or its E-DII in the included studies followed these sequential steps: (1) Dietary Data Acquisition: Individual dietary intake data were collected using study-specific, validated Food Frequency Questionnaires (FFQs) or dietary records. These instruments captured the habitual consumption frequency and portion sizes of a wide range of food items. (2) Linkage to a Global Reference Database: The intake amounts of food parameters (nutrients and other bioactive food components) for each individual were linked to a standardized global reference database. This database provides the mean intake and standard deviation for each of up to 45 pro- and anti-inflammatory dietary components (e.g., carbohydrates, fats, proteins, fiber, vitamins, minerals, flavonoids, caffeine) based on dietary consumption patterns from 11 populations around the world. (3) Calculation of Z-scores and Centering: For each dietary parameter, a Z-score was calculated by subtracting the “global mean” intake from the individual’s reported intake and then dividing this difference by the global standard deviation. To minimize the effect of “right skewing” (a common characteristic of dietary data), this Z-score was then converted to a percentile score and centered by doubling the percentile and subtracting 1. (4) Multiplication by the Inflammatory Effect Score: The centered percentile value for each parameter was multiplied by its corresponding “inflammatory effect score.” This score is a literature-derived weight assigned to each dietary component, based on a systematic review of nearly 2,000 peer-reviewed articles examining the component’s effect on six specific inflammatory biomarkers: interleukin (IL)-1β, IL-4, IL-6, IL-10, tumor necrosis factor (TNF)-α, and C-reactive protein (CRP). The score is positive for pro-inflammatory components, negative for anti-inflammatory components, and zero for components with no robust evidence of an inflammatory effect. (5) Aggregation to Create the Overall DII Score: The weighted values for all assessed dietary parameters were summed to yield the overall DII score for each participant. A higher DII score indicates a more pro-inflammatory dietary pattern, while a lower (or negative) score indicates a more anti-inflammatory dietary pattern. (6) Energy Adjustment (for E-DII): To account for the potential confounding effect of total energy intake, many studies calculated the E-DII. This was typically achieved using the nutrient density method, where the intake of each dietary component was expressed per 1,000 kilocalories (or 4,184 kilojoules) of total energy intake before performing steps 2–5. This approach isolates the effect of diet composition from the effect of total caloric consumption.

The specific number of dietary components used to calculate the DII varied across studies (commonly ranging from 30 to 45 items), depending on the comprehensiveness of the FFQ and data availability. However, all included studies adhered to the core computational framework described above, ensuring comparability of the DII construct across different populations and study designs.

### 2.5 Study selection

The literature screening process was carried out independently by two reviewers to minimize bias. In the initial phase, all retrieved records underwent title and abstract screening to eliminate clearly ineligible publications. The remaining potentially relevant articles then proceeded to full-text assessment for eligibility determination. Any disagreements between the reviewers at either stage were resolved through consensus discussion or, when necessary, by arbitration from a senior researcher.

A standardized, pre-piloted data extraction form was utilized to systematically collect information from the included studies. The following data domains were extracted: (1) General study attributes: including primary author, year of publication, study name, geographical location, research design, and follow-up period where applicable. (2) Participant characteristics: sample size (number of cases/controls or cohort participants), age, menopausal status, etc. (3) Exposure and outcome assessment: method of DII/E-DII calculation (based on specific dietary assessment tool), comparison categories (e.g., highest vs. lowest quantile), fully adjusted effect estimates (HR, OR with their 95% CI). (4) Covariate adjustment: potential confounding factors adjusted for in the statistical models (e.g., age, BMI, energy intake, alcohol consumption, smoking status, physical activity, menopausal status, family history).

### 2.6 Quality assessment (risk of bias)

Two reviewers independently assessed the methodological quality of the included cohort and case-control studies using the Newcastle-Ottawa Scale (NOS), as recommended by the Agency for Healthcare Research and Quality (AHRQ). The NOS evaluates studies across three domains: (1) selection of study groups, (2) comparability of groups, and (3) ascertainment of either the exposure (for case-control studies) or outcome (for cohort studies). A star system is used to award points within each domain, with a maximum possible score of 9 stars, indicating the highest methodological quality. Disagreements in quality assessment were resolved by consensus.

### 2.7 Statistical analysis

(1)    *Effect size pooling*: For dichotomous outcomes (e.g., disease risk, mortality risk), fully adjusted ORs or HRs with their 95% CIs were extracted and pooled. Effect sizes comparing different DII/E-DII quantiles (e.g., Q4 vs. Q1) were considered the primary analysis. If studies only provided effect estimates for DII as a continuous variable, the OR/HR corresponding to a one-unit increase in the DII score was pooled. A random-effects model (DerSimonian and Laird method) was used to pool effect sizes, accounting for potential heterogeneity among studies.
(2) *Assessment of heterogeneity*: The degree of variability among the included studies was quantified using Cochrane’s Q test (with a significance threshold of α = 0.10) and the I^2^ statistic. The magnitude of heterogeneity was categorized as follows: I^2^ values of 25%, 50, and 75% represented low, moderate, and high heterogeneity, respectively. A random-effects model was implemented for all pooled analyses to account for expected methodological and population variations.
(3) *Subgroup and sensitivity analyses*: Pre-specified subgroup analyses were conducted to investigate potential effect modifiers, including study design (cohort versus case-control) and menopausal status (premenopausal versus postmenopausal). Additional sensitivity analyses were performed to evaluate the robustness of the primary findings.
(4) *Evaluation of publication bias*: Potential publication bias was examined through visual inspection of funnel plots for analyses including at least 10 studies, as the statistical power of such tests is limited with a smaller number of studies. For analyses with fewer studies, publication bias was not formally assessed due to low test power. When presented, asymmetry in the funnel plot was interpreted as suggestive of possible publication bias, acknowledging that other factors (e.g., heterogeneity, methodological differences) could also cause asymmetry.
(5) *Dose-response analysis*: For studies that reported the DII as a continuous variable, the adjusted OR or HR for a one-unit increase in the DII score was directly extracted and pooled. These estimates were derived from regression models (logistic regression for case-control studies, Cox proportional hazards regression for cohort studies) that treated DII as a continuous linear predictor, adjusting for relevant confounders. For studies that reported DII in categories (e.g., quartiles) and did not provide a per-unit estimate, the continuous effect size could not be derived. The pooled effect per one-unit DII increment was therefore calculated by synthesizing the estimates from studies that provided continuous data, using the random-effects model described above.

All statistical computations were executed using R software (version 4.2.0) with the “metafor” package. Statistical significance was defined as a two-tailed *P*-value of less than 0.05.

## 3 Results

### 3.1 Study selection process

**FIGURE 1:**
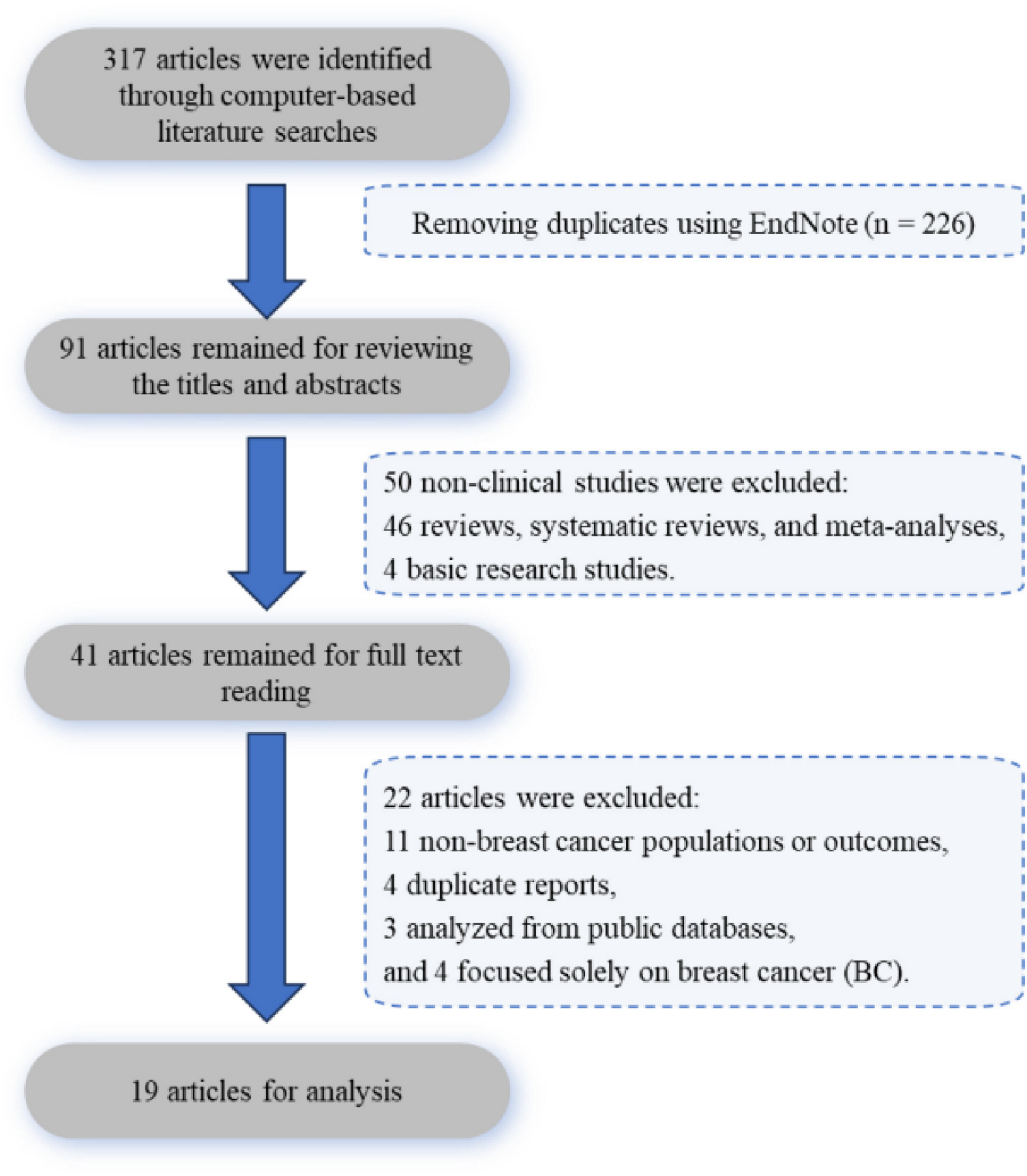
Literature screening and selection flow diagram.

The initial electronic database search identified 317 records. After removing duplicates using EndNote, 91 unique records remained. Screening of titles and abstracts led to the exclusion of 50 non-clinical studies, which comprised 46 reviews, systematic reviews, and meta-analyses, and 4 basic science studies. The full texts of the remaining 41 articles were assessed for eligibility. This resulted in the exclusion of 11 studies with non-BC populations or outcomes, 4 duplicate reports, 3 analyses from public databases, and 4 studies that did not exclusively focus on BC. Consequently, 19 studies were included in the final analysis (Figure 1).

### 3.2 Characteristics of included studies

A total of 19 studies were included. Among them, 14 case-control studies involved approximately 19,000 BC patients and 24,000 controls, while 5 prospective cohort studies encompassed approximately 280,000 female participants. The studies utilized different Food Frequency Questionnaires (FFQs), and the methods for categorizing the DII varied, including dichotomization, tertiles, quartiles, and quintiles. Geographically, Iran contributed the most studies, followed by the United States. Regarding methodological quality, the NOS scores ranged from 7 to 8, indicating that all included studies were of moderate to high quality (Table 1).

**TABLE 1:** Basic characteristics of the included studies.

References	Region	Enrollments	FFQ	DII	NOS scores	OR/HR with 95%CI (highest vs. lowest)	Key adjustments
**Case-control (*n* = 14)**							
Ge et al. (19)	German	2887 postmenopausal breast cancer patients and 5512 healthy age-matched controls	176	Q5	7	1.01 (0.86–1.17)	Age, energy intake, hormone therapy
Huang et al. (10)	China	867 breast cancer patients and 824 healthy age-matched controls (premenopausal and postmenopausal)	81	Q4	8	2.28 (1.71, 3.03)	Age, BMI, energy intake
Vahid et al. (12)	Iran	145 breast cancer patients and 148 healthy age-matched controls	168	Q3	7	6.94 (3.26, 14.79)	Age, physical activity, smoking
Lee et al. (14)	Korean	364 breast cancer patients and 364 age-matched controls (premenopausal and postmenopausal)	106	Q3	7	3.68 (2.34, 5.8)	Age, smoking, BMI
Obón-Santacana et al. (20)	Spain	1567 breast cancer cases and 1486 age sex and region matched controls (premenopausal and postmenopausal)	140	Q3	8	1.22 (0.99, 1.52)	Age, physical activity, BMI
Niclis et al. (21)	Argentina	317 breast cancer patients and 526 age-matched controls	127	Q3	6	1.34 (1.05, 1.70)	Age, BMI, energy intake
Gholamalizadeh et al. (22)	Iran	180 breast cancer patients and 360 healthy age-matched controls	168	Q2	7	5.02 (1.43, 17.58)	Age, physical activity, family history
Hammad et al. (23)	Jordan	200 breast cancer patients and 200 healthy age income, and marital status-matched controls	122	Q3	7	1.11 (0.61, 2.01)	Age, smoking, energy intake
Jalali et al. (11)	Iran	136 breast cancer patients and 272 healthy age-matched controls (premenopausal and postmenopausal)			6	2.64 (1.12, 6.25)	Age, BMI, Energy intake
Hajji-Louati et al. (24)	France	872 breast cancer cases and 966 population controls (premenopausal and postmenopausal)	153	Q4	7	1.31 (1.00, 1.73)	Age, physical activity, hormone therapy
Sasanfar et al. (13)	Iran	412 women with pathologically confirmed breast cancer and 456 age-matched apparently healthy controls	168	Q4	8	1.43 (0.94, 2.18)	Age, energy intake, physical activity
Hayati et al. (25)	Iran	1007 women with histopathologically confirmed BrCA and 1004 age-matched controls	136	Q4	7	1.87 (1.42, 2.47)	Age, physical activity, energy intake
Sohouli et al. (26)	Iran	253 patients with BrCa and 267 non-BrCa controls	168	Q4	7	2.17 (1.12, 4.22)	Age, physical activity, BMI
Ghanbari et al. (27)	Iran	150 age-matched women with newly diagnosed breast cancer and controls	147	Q4	8	2.38 (1.23, 4.59)	Age, BMI
**Cohort (*n* = 5)**							
Shivappa et al. (9)	American	1,986 women ages 55–69 years	121	Q3	8	1.11 (1.00, 1.22)	Age, energy intake, BMI
Tabung et al. (4)	American	122,788 postmenopausal women with no prior cancer	122	Q5	8	0.99 (0.91, 1.07)	Age, energy intake, physical activity
Hajji-Louati et al. (15)	France	67,879 women with no prior cancer	–	Q5	8	1.13 (1.04, 1.23)	Age, energy intake, BMI
Shivappa et al. (16)	American	49,258 women who returned a completed comprehensive questionnaire in 1991–1992	80	Q4	8	1.18 (1.00, 1.39)	Age, physical activity, BMI
Park et al. (28)	American	43,563 Sister Study cohort participants at enrollment in 2003–2009	110	Q4	7	1.00 (0.96, 1.04)	Age, physical activity, BMI

BC, breast cancer; BMI, body mass index (kg/m^2^); DII, Dietary Inflammatory Index; E-DII, energy-adjusted DII; FFQ, Food Frequency Questionnaire; HR, hazard ratio; IWHS, Iowa Women’s Health Study; NOS, Newcastle-Ottawa Scale; OR, odds ratio; WHI-OS, Women’s Health Initiative Observational Study; WLH, Women’s Lifestyle and Health.

The detailed scoring across the three NOS domains for each study is summarized in Table 2. The total NOS scores ranged from 6 to 8, with a median score of 7. Fourteen studies (73.7%) scored 7 or above, which is conventionally considered indicative of moderate to high quality. The most common strengths across studies were adequate case definition and ascertainment of exposure. Variability in scores was primarily observed in the “Comparability” domain, reflecting differences in the extent of adjustment for potential confounders (e.g., some studies adjusted only for age and energy intake, while others included a more comprehensive set of covariates such as BMI, physical activity, and reproductive factors).

**TABLE 2:** Detailed methodological quality assessment of included studies using the Newcastle-Ottawa Scale (NOS).

References	Study design	Selection (max: 4) ★	Comparability (max: 2) ★	Exposure/ outcome (max: 3) ★	Total score (max: 9) ★
**Case-control studies**					
Ge et al. (19)	Case-control	★★★★	★	★★	7
Huang et al. (10)	Case-control	★★★★	★★	★★	8
Jalali et al. (11)	Case-control	★★★	★	★★	6
Vahid et al. (12)	Case-control	★★★	★★	★★	7
Lee et al. (14)	Case-control	★★★	★★	★★	7
Obón-Santacana et al. (20)	Case-control	★★★★	★★	★★	8
Niclis et al. (21)	Case-control	★★★	★	★★	6
Hammad et al. (23)	Case-control	★★★	★★	★★	7
Hajji-Louati et al. (24)	Case-control	★★★	★★	★★	7
Sasanfar et al. (13)	Case-control	★★★★	★★	★★	8
Hayati et al. (25)	Case-control	★★★	★★	★★	7
Gholamalizadeh et al. (22)	Case-control	★★★	★★	★★	7
Sohouli et al. (26)	Case-control	★★★	★★	★★	7
Ghanbari et al. (27)	Case-control	★★★★	★★	★★	8
**Cohort studies**					
Shivappa et al. (16)	Cohort	★★★★	★★	★★	8
Tabung et al. (4)	Cohort	★★★★	★★	★★	8
Shivappa et al. (9)	Cohort	★★★★	★★	★★	8
Park et al. (28)	Cohort	★★★	★★	★★	7
Hajji-Louati et al. 2023 (15)	Cohort	★★★★	★★	★★	8

Selection (Case-control): Adequacy of case definition, representativeness of cases, selection and definition of controls. Selection (Cohort): Representativeness of exposed cohort, selection of non-exposed cohort, ascertainment of exposure, demonstration that outcome was absent at start. Comparability: Design or analysis controls for age and energy intake (★), and additionally for other key factors (e.g., BMI, physical activity) (★). Exposure (Case-control): Secure ascertainment of exposure, same method for cases/controls, non-response rate. Outcome (Cohort): Independent/record-based assessment, follow-up length adequate, completeness of follow-up.

### 3.3 Meta-analysis results

#### 3.3.1 Results from case-control studies

First, we investigated the relationship between DII and BC risk in case-control studies. Given the varying methods of DII data handling across studies—including dichotomization, tertiles, quartiles, quintiles, and treatment as a continuous variable—we analyzed the association by comparing the highest DII category against the lowest reference category. Subgroup analyses were performed based on the menopausal status of the participants: overall, premenopausal, and postmenopausal.

**FIGURE 2:**
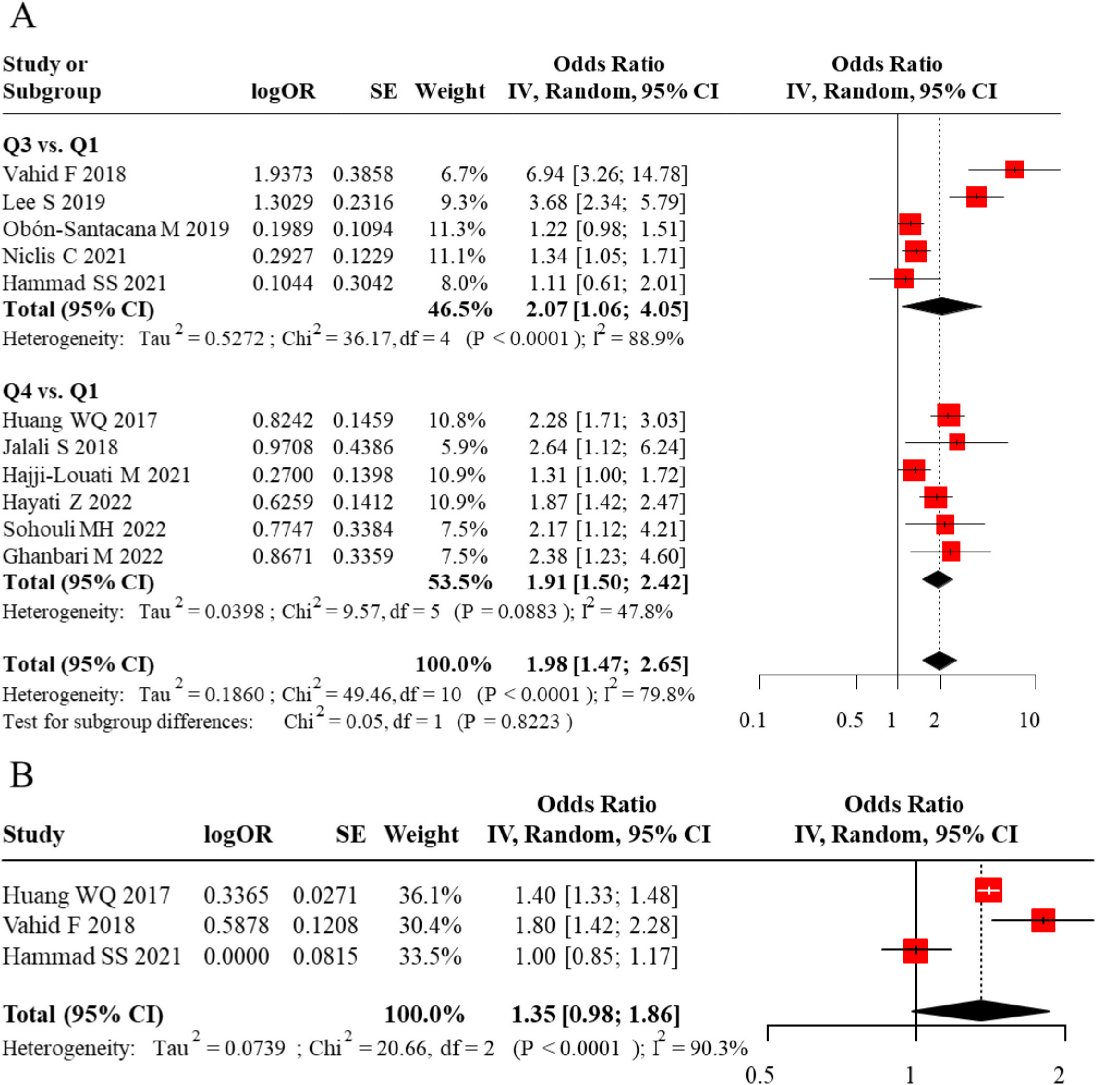
**(A)** Forest plot comparing the odds ratios (OR) for DII in Q3 vs. Q1 and Q4 vs. Q1 groups. **(B)** Pooled odds ratio (OR) for DII as a continuous variable, with heterogeneity statistics.

**FIGURE 3:**
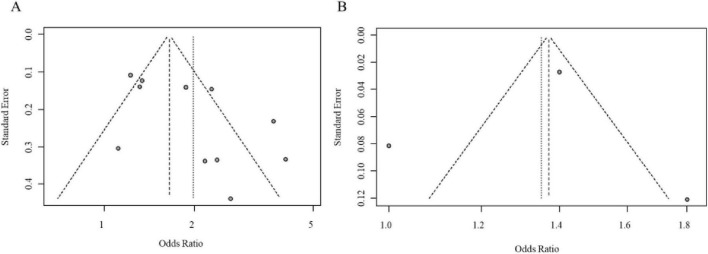
**(A)** Funnel plot assessing publication bias for the meta-analysis of DII and breast cancer risk in case-control studies. **(B)** Further confirmation of no significant publication bias in the meta-analysis.

Initially, we analyzed data from populations with unspecified menopausal status. Eleven studies reported on the DII-BC relationship in these mixed populations: 5 studies presented results using DII tertiles, 6 used quartiles, and 3 reported DII as a continuous variable. The analysis revealed that a higher DII (comparing the highest vs. lowest category) was significantly associated with an increased risk of BC when DII was analyzed by tertiles (pooled adjusted OR = 2.07, 95% CI = 1.06–4.05) and by quartiles (pooled adjusted OR = 1.91, 95% CI = 1.50–2.42). However, when DII was treated as a continuous variable, the association was not statistically significant. Notably, significant heterogeneity was observed for both categorical approaches. The forest plot for the meta-analysis of DII and BC risk in case-control studies (unspecified menopausal status) is shown in Figure 2. Visual inspection of the funnel plot suggested approximate symmetry (Figure 3). However, given the limited number of studies in this analysis, the power to detect publication bias was low, and this result should be interpreted with caution.

**FIGURE 4:**
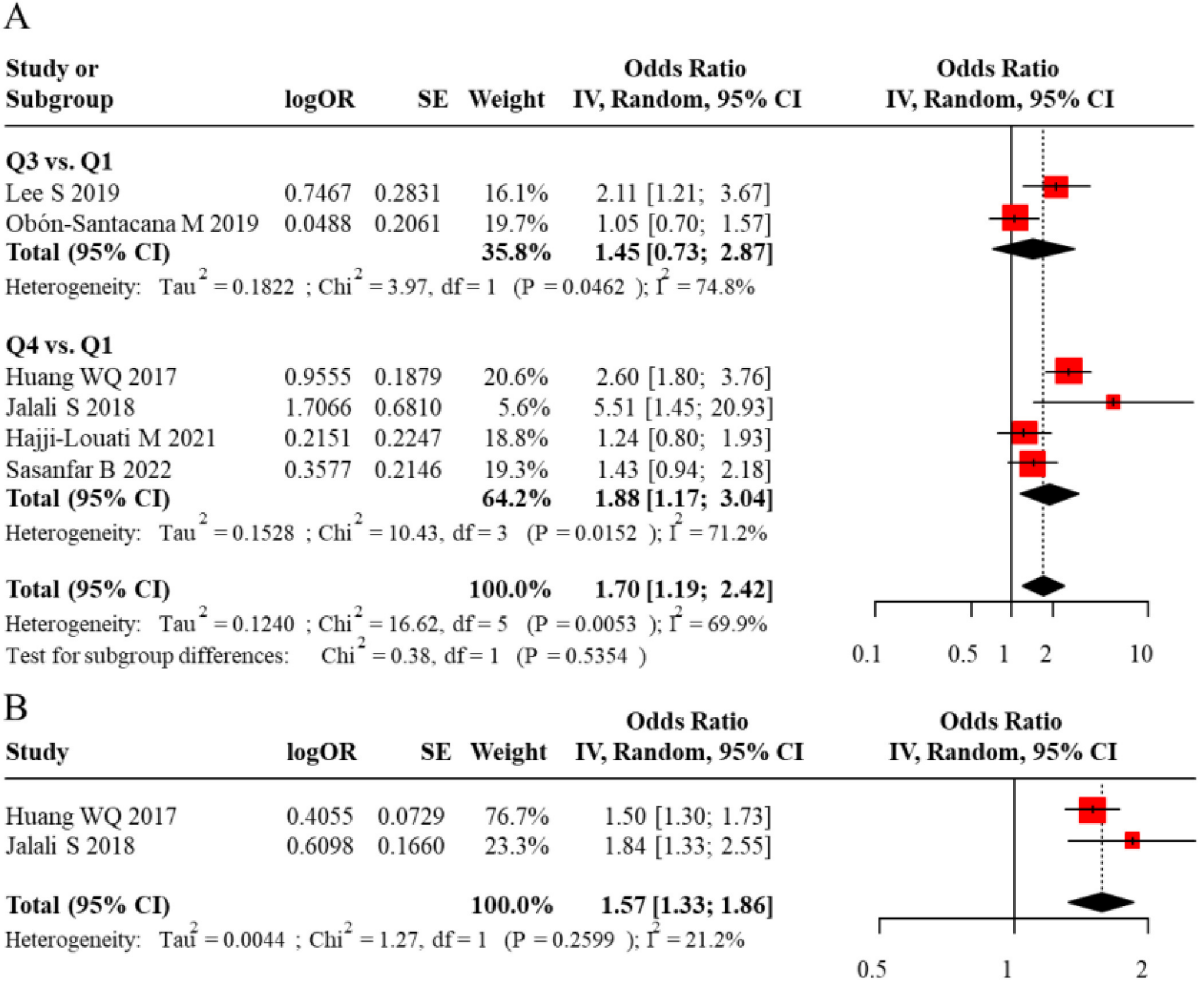
**(A)** Forest plot comparing the odds ratios (OR) for DII in premenopausal women from case-control studies. **(B)** Pooled odds ratio (OR) for DII as a continuous variable in premenopausal women, with heterogeneity statistics.

**FIGURE 5:**
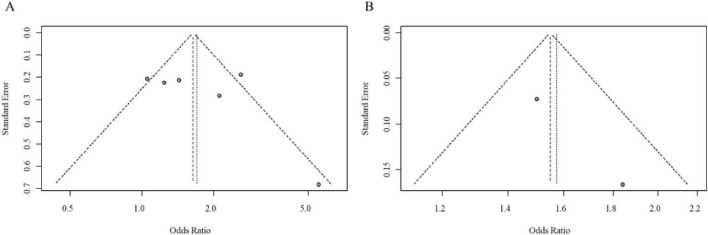
**(A)** Funnel plot assessing publication bias for the meta-analysis of DII and breast cancer risk in premenopausal women. **(B)** Further confirmation of no significant publication bias in premenopausal women.

Next, we analyzed data from premenopausal women. Six studies reported on this subgroup: 2 used DII tertiles, 4 used quartiles, and 2 provided data for DII as a continuous variable. The analysis showed that a higher DII was not a significant risk factor for BC when analyzed by tertiles (pooled adjusted OR = 1.45, 95% CI = 0.73–2.87). However, a significant association was observed when DII was analyzed by quartiles (pooled adjusted OR = 1.88, 95% CI = 1.17–3.04) and as a continuous variable (pooled adjusted OR = 1.57, 95% CI = 1.33–1.86) (Figure 4). The funnel plot appeared largely symmetrical, suggesting no substantial publication bias (Figure 5).

**FIGURE 6:**
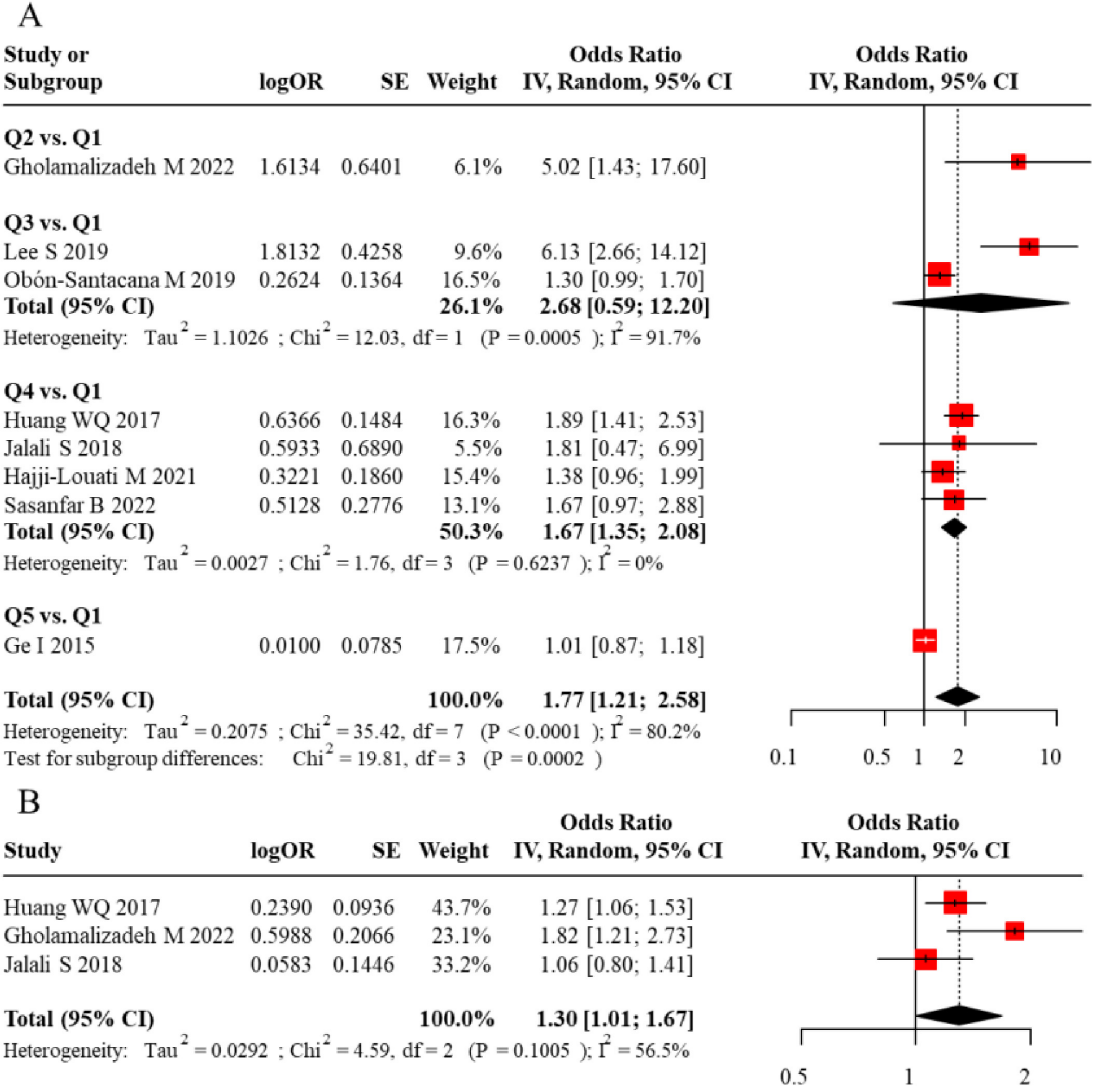
**(A)** Forest plot comparing the odds ratios (OR) for DII in postmenopausal women from case-control studies. **(B)** Pooled odds ratio (OR) for DII as a continuous variable in postmenopausal women, with heterogeneity statistics.

**FIGURE 7:**
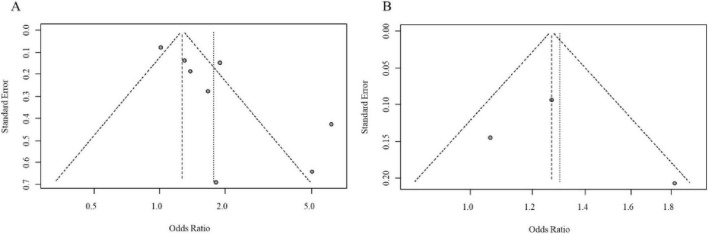
**(A)** Funnel plot assessing publication bias for DII and BC risk in postmenopausal women. **(B)** Further confirmation of no significant publication bias in the meta-analysis.

Subsequently, we analyzed data from postmenopausal women. Seven studies reported on this subgroup: 1 study used DII dichotomization, 2 used tertiles, 4 used quartiles, and 3 provided data for DII as a continuous variable. The results indicated that a higher DII was a significant risk factor for BC when analyzed by dichotomization (pooled adjusted OR = 5.02, 95% CI = 1.43–17.60) and by quartiles (pooled adjusted OR = 1.67, 95% CI = 1.35–2.08). In contrast, when DII was modeled using tertile or quintile groupings, the pooled analyses did not reveal statistically significant associations with breast cancer risk (tertiles: adjusted OR = 2.68, 95% CI 0.59–12.20; quintiles: adjusted OR = 1.01, 95% CI 0.87–1.18). However, analysis treating DII as a continuous exposure variable demonstrated a statistically significant positive association, with each unit increase in DII corresponding to a 77% elevation in breast cancer risk (adjusted OR = 1.77, 95% CI 1.21–2.58). The corresponding forest plot for these analyses is presented in Figure 6. Evaluation of publication bias through funnel plot inspection revealed generally symmetrical distribution of effect estimates, indicating minimal evidence of publication bias (Figure 7).

#### 3.3.2 Results from cohort studies

**FIGURE 8:**
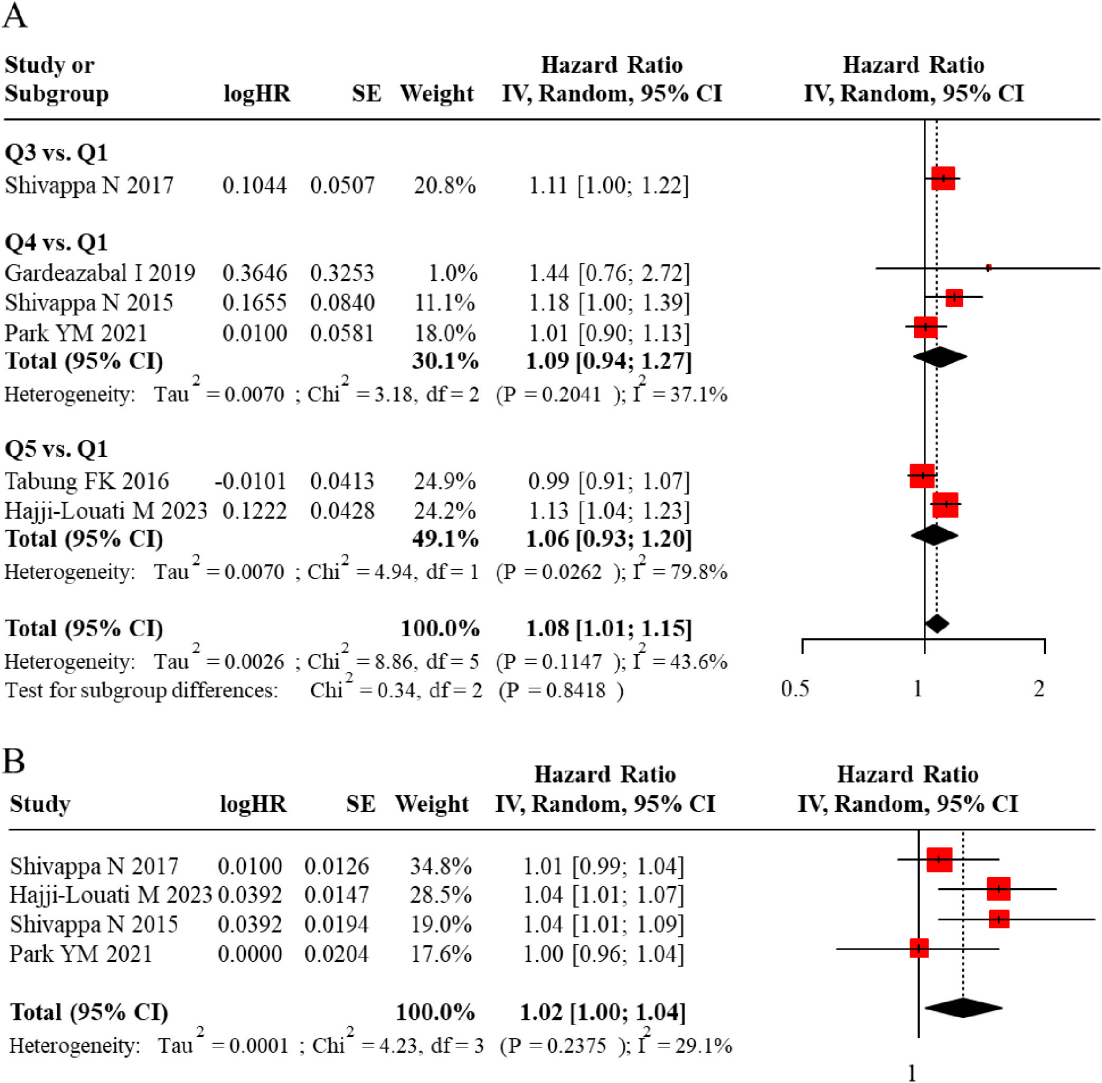
**(A)** Forest plot showing the association between DII and BC risk in cohort studies. **(B)** Comparison of highest vs. lowest DII quartiles in the cohort study meta-analysis.

**FIGURE 9:**
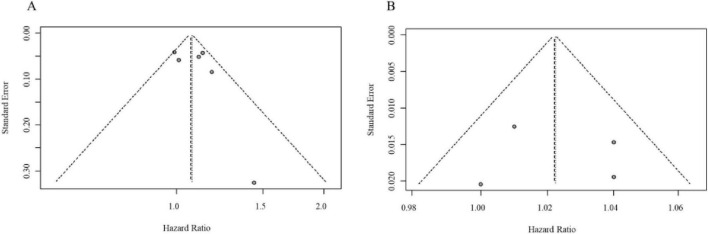
**(A)** Funnel plot assessing publication bias for DII and BC risk in cohort studies. **(B)** Further confirmation of no significant publication bias in the cohort study analysis.

In the cohort studies, all participants were free of BC at baseline, and the analysis focused on BC risk during follow-up according to different DII levels. Pooled analysis revealed a significantly elevated hazard of breast cancer among participants in the highest DII category relative to the lowest (adjusted HR = 1.08, 95% CI: 1.01–1.15). Consistently, a dose-response relationship was observed when modeling DII continuously, with each per-unit increment in the DII score corresponding to a 2% increase in breast cancer risk (adjusted HR = 1.02, 95% CI: 1.00–1.04). The forest plot for the cohort study meta-analysis is presented in Figure 8. The funnel plot appeared broadly symmetrical, indicating no obvious publication bias (Figure 9).

## 4 Discussion

This systematic review and meta-analysis, synthesizing evidence from 19 observational studies (14 case-control and 5 prospective cohort studies), confirms a positive association between the DII and BC risk. In case-control studies, a higher DII (comparing the highest vs. lowest quantile) was significantly associated with an increased risk of BC, and this association was observed in both premenopausal and postmenopausal women. Prospective cohort studies further validated that individuals with a higher DII had a significantly greater risk of BC compared to those with a lower DII, with each 1-unit increase in the DII score corresponding to an approximate 2% increase in risk. These results align with converging evidence from contemporary epidemiological research, thereby corroborating the hypothesis that dietary patterns with pro-inflammatory properties contribute to the etiology of breast cancer.

In our analysis of case-control studies with populations of unspecified menopausal status, we observed a notable discrepancy: the association between DII and breast cancer risk was statistically significant when DII was analyzed categorically (by tertiles or quartiles) but not when modeled as a continuous variable. This pattern suggests several possibilities. First, it may reflect limited statistical power in the continuous analysis, as fewer studies (*n* = 3) provided effect estimates for a per-unit DII increase compared to those using categorical comparisons. Second, the relationship between DII and breast cancer risk in this heterogeneous group may not be strictly linear across the entire range of DII scores. Categorical analysis can capture non-linear or threshold effects by comparing extremes (e.g., highest vs. lowest quartile), whereas a continuous linear model assumes a uniform risk increase per unit, which might not hold true if the risk escalates only beyond a certain inflammatory threshold. Third, the grouping of pre- and postmenopausal women, who have distinct hormonal milieus and potentially different diet-cancer pathways, into a single “unspecified” category could have introduced unmeasured confounding and effect modification, obscuring a clear linear trend when analyzed continuously. This finding underscores the importance of menopausal stratification in future studies and suggests that the risk attributable to a pro-inflammatory diet might be more readily detectable when comparing individuals with substantially different dietary patterns, rather than assuming a simple linear gradient.

The results of this study align closely with research conducted across diverse global regions and populations. A systematic review by Lamchabbek et al. focusing on the Middle East and North Africa region reported a significant positive association between a high DII (pro-inflammatory diet) and BC risk (29). It suggested that dietary patterns characteristic of the region, such as high intake of milk and white bread, might exacerbate risk by enhancing the dietary inflammatory potential, whereas the consumption of anti-inflammatory foods like fruits, vegetables, and fish could mitigate it. This aligns perfectly with our core finding that a high DII increases BC risk. On a metabolic level, a prospective study by Long et al. based on data from over 270,000 women in the UK Biobank found that a high DII was associated with elevated levels of 26 pro-inflammatory-related metabolites (e.g., lipoproteins, saturated fatty acids, ketone bodies) (30). This metabolic signature was associated with a 21% increased risk of BC (HR = 1.21, 95% CI = 1.01–1.46), with increased proportions of saturated fatty acids and acetone concentration identified as key metabolic markers for heightened risk. This provides direct evidence for “pro-inflammatory diet mediating BC occurrence through metabolic disturbances” and explains the biological plausibility of the DII-risk association observed in our study. While our overall conclusion aligns with that of the earlier meta-analysis by Chen et al. (8), the present study offers several critical updates and extensions. First, and most importantly, our review includes literature published up to September 2025, thereby incorporating five additional years of evidence. This update nearly doubles the number of available case-control studies (from approximately 8 in Chen et al.’s analysis to 14 in ours) and adds more recent, large-scale cohort data [e.g., (15)]. Second, our analysis provides a more granular examination of the dose-response relationship. We were able to quantify that each one-unit increase in the DII is associated with an approximately 2% increase in breast cancer risk in prospective cohorts, an estimate not reported in the previous meta-analysis. Third, we performed extensive subgroup analyses stratified by menopausal status within case-control studies, revealing nuanced patterns: for example, a significant association was observed in postmenopausal women when DII was analyzed as a continuous variable or by quartiles, but not when using tertiles or quintiles—a finding highlighting the impact of categorization methods, which was not explored in depth previously. Fourth, our updated search allowed for a broader geographical representation, particularly strengthening the evidence from Middle Eastern populations, which were less represented in earlier syntheses. Finally, we conducted a comprehensive evaluation of methodological heterogeneity, explicitly discussing how variations in FFQ instruments and DII categorization strategies may act as confounders, a methodological critique that advances the interpretation of pooled estimates in this field.

The biological mechanisms underlying the DII-BC association primarily revolve around the central role of chronic inflammation in cancer development and progression. Chronic inflammation drives tumorigenesis by inducing oxidative stress, causing DNA damage, promoting cell proliferation, inhibiting apoptosis, stimulating angiogenesis, and activating signaling pathways involved in tumorigenesis, such as NF-κB and JAK-STAT pathways (3). The DII score correlates positively with circulating levels of various inflammatory markers, such as C-reactive protein (CRP), tumor necrosis factor-alpha (TNF-α), and interleukin-6 (IL-6), which have been implicated in increased BC risk and poor prognosis (31, 32). Furthermore, pro-inflammatory diets are typically rich in red meat, processed meat, refined carbohydrates, and saturated fats, while lacking anti-inflammatory components like fruits, vegetables, whole grains, and omega-3 polyunsaturated fatty acids. This dietary pattern may collectively foster a microenvironment conducive to tumor initiation and progression through various mechanisms, including altering gut microbiota, inducing insulin resistance, and promoting adipose tissue inflammation (9). The metabolomics investigation conducted by Long et al. elucidated a distinct metabolic profile linked to high DII scores, characterized by an elevated proportion of saturated fatty acids and a concurrent reduction in fatty acid unsaturation (30). Saturated fatty acids can exacerbate inflammatory responses by activating the TLR4/MyD88 pathway while simultaneously inhibiting the AMPK pathway, promoting fat accumulation. The subsequent secretion of leptin by adipose tissue further enhances inflammation (Boyer et al., (33), found a positive correlation between high DII and leptin levels (33)), creating a vicious cycle of “pro-inflammatory diet → metabolic disturbance → exacerbated inflammation.” Pro-inflammatory diets may also compromise the body’s anti-tumor immunity by reducing antioxidant capacity and immune cell activity. Research by Alkan et al. showed that the dietary total antioxidant capacity (dTAC) was significantly lower in BC patients compared to healthy controls, and a high DII was negatively correlated with dTAC (34). This may lead to decreased reactive oxygen species (ROS) scavenging capacity, subsequently causing DNA damage and impairing the anti-tumor functions of T cells and NK cells.

Some heterogeneity was observed in this study, which can be explained by three main sources. First, and most critically, the included studies employed inconsistent methods for categorizing the continuous DII score—including dichotomization, tertiles, quartiles, and quintiles. This lack of standardization acts as a methodological confounder that directly impacts the magnitude of the reported effect estimates. For instance, in our analysis of postmenopausal women, dichotomization (maximizing contrast) showed a significantly elevated risk (OR = 5.02, 95% CI = 1.43–17.60), whereas quintile analysis (a finer categorization) found no significant association (OR = 1.01, 95% CI = 0.87–1.18). This illustrates how the choice of cut-points can either amplify or dilute the observed between-group risk difference. Similarly, research by Ng et al. indicated that associations involving the DII can vary depending on the categorization method, underscoring the need for standardized approaches in future studies (35). Second, the varying number of items in the Food Frequency Questionnaires (FFQs) used across studies (ranging from 81 to 176 items) may have affected the precision of DII calculation. While all instruments were validated, more comprehensive FFQs might better capture the diet’s inflammatory potential, whereas shorter ones could introduce measurement error and attenuate the true association. Third, the included populations spanned diverse geographical regions, including Asia, Europe, the Middle East, and the Americas. Differences in underlying dietary patterns, such as the more pro-inflammatory diets noted in some Iranian populations (11) compared to the potentially mitigating Mediterranean pattern in Europe (36), could influence the strength of the DII-BC association. Additionally, variability in the extent of adjustment for key confounders (e.g., obesity, hormone use) across studies might contribute to residual confounding and heterogeneity in the pooled estimates.

This study has several limitations. First, the vast majority of included studies were observational. Although we adjusted for known confounders where possible, the influence of residual or unmeasured confounding cannot be entirely ruled out. Second, and inherent to nutritional epidemiology, most included studies assessed diet using a FFQ at a single time point. While FFQs are designed to capture habitual intake, a single assessment may not account for long-term dietary changes and is subject to recall bias and measurement error. Critically, such non-differential measurement error in exposure assessment typically biases the observed effect estimates toward the null (attenuation bias). Therefore, the true association between a pro-inflammatory diet and breast cancer risk might be stronger than our pooled estimates suggest. Third, the methodological variations discussed above, particularly in DII categorization and FFQ design, represent inherent limitations of the available literature that our analysis could not overcome, contributing to the observed statistical heterogeneity. Fourth, despite our comprehensive search strategy, potential publication bias (wherein positive results are more likely to be published) might still be present. Fifth, while we performed funnel plot inspections for some analyses, the statistical power to detect publication bias was limited due to the modest number of studies included in several subgroup analyses. Therefore, we cannot definitively rule out the possibility of publication bias. Sixth, regarding the generalizability of our findings, while our analysis included studies from Asia, Europe, the Middle East, and the Americas, the evidence base is not uniformly distributed across global populations. A notable concentration of studies originated from Iran and the United States. Consequently, our pooled estimates may most accurately reflect the DII-breast cancer association within populations characterized by Western or Middle Eastern dietary patterns. The applicability of these findings to regions with fundamentally different dietary cultures, such as Sub-Saharan Africa or Southeast Asia, where staple foods, food processing, and overall dietary composition differ substantially, remains uncertain and warrants investigation in future studies. Moreover, there was inconsistency in the adjustment for key confounding variables across the included studies. While total energy intake—a critical confounder for any dietary index—was adjusted for in nearly 90% of studies, adjustment for physical activity was not universal (approximately 74%). This incomplete adjustment for a major behavioral risk factor could lead to residual confounding, meaning that the observed association between DII and breast cancer risk might be partially attributed to differences in physical activity levels rather than diet alone. Finally, this study primarily focused on the DII as a composite index and did not delve into the independent effects of specific foods or nutrients on BC risk and prognosis. Investigating these specific dietary components represents a potential direction for future research.

## 5 Conclusion

Our meta-analysis supports a positive association between higher DII scores, indicative of a pro-inflammatory dietary pattern, and an increased risk of breast cancer among women. This relationship was most evident among postmenopausal women. This association was observed despite moderate statistical heterogeneity across studies, which likely stems from methodological variations in dietary assessment and DII categorization. These findings underscore the important role of chronic inflammation in the etiology of BC and provide a solid evidence-based foundation for utilizing dietary interventions in the primary prevention and secondary management of BC. Future research should focus on conducting large-scale, long-term randomized controlled trials to verify the causal effect of anti-inflammatory dietary interventions on reducing BC risk and to further elucidate the underlying biological mechanisms, particularly concerning different molecular subtypes and genetic backgrounds of BC. Concurrently, developing more precise and convenient DII assessment tools and integrating them into clinical practice and public health strategies hold profound significance for reducing the global burden of breast cancer.
